# Association between socioeconomic position and diabetic foot ulcer outcomes: a population-based cohort study in South Korea

**DOI:** 10.1186/s12889-021-11406-3

**Published:** 2021-07-14

**Authors:** Jeong Hyun Ha, Heejin Jin, Ji-Ung Park

**Affiliations:** 1 Department of Plastic and Reconstructive Surgery, Seoul National University Boramae Hospital, Seoul National University College of Medicine, Seoul, 07061 Republic of Korea; 2 Medical Research Collaborating Center, Seoul Metropolitan Government–Seoul National University Boramae Medical Center, Seoul, 07061 South Korea

**Keywords:** Diabetic foot ulcer, Socioeconomic position, Amputation, Mortality

## Abstract

**Background:**

Low socioeconomic position (SEP) is associated with a high incidence of diabetic foot ulcers (DFUs). However, reports on the association between SEP and DFU outcomes are limited. Therefore, in this study, we investigated this association and determined the prognostic factors of DFU outcomes.

**Methods:**

The total cohort comprised 976,252 individuals. Using probability sampling, we randomly selected a sample of patients by reviewing the data from the Health Insurance Review and Assessment Service database of South Korea during 2011–2015. Residence, household income, and insurance type represented SEP. The primary outcome was amputation, and the secondary outcome was mortality. A multivariate model was applied to identify the predictive factors. Amputation-free survival and overall survival were calculated using the Kaplan-Meier method.

**Results:**

Among 976,252 individuals in the cohort, 1362 had DFUs (mean age 62.9 ± 12.2 years; 42.9% were women). Overall amputation and mortality rates were 4.7 and 12.3%, respectively. Male sex (hazard ratio [HR], 2.41; *p* < 0.01), low SEP (HR 5.13, 5.13; *p* = 0.018), ophthalmopathy (HR, 1.89; *p* = 0.028), circulatory complications (HR, 2.14; *p* = 0.020), and institutional type (HR, 1.78; *p* = 0.044) were prognostic factors for amputation. Old age (HR, 1.06; *p* < 0.01), low SEP (HR, 2.65; *p* < 0.01), ophthalmopathy (HR, 1.74; *p* < 0.01), circulatory complications (HR, 1.71; *p* < 0.01), and institution type (HR 1.84; *p* < 0.01) were predictors of mortality.

**Conclusions:**

DFU patients with a low SEP are strongly associated with increased amputation and mortality rates. Along with age and comorbidities, SEP could provide the basis for risk assessment of adverse outcomes in DFU. Providing targeted care for this population considering SEP may improve the prognosis.

## Background

Diabetic foot ulcers (DFUs) are one of the major complications of neuropathy and microvascular disease in patients with diabetes. The annual incidence and prevalence rates of DFU are 1–4% and 5–10%, respectively, while the lifetime risk of developing DFUs is 15% in diabetic patients [1, 2]. DFUs, the major cause of nontraumatic amputation, are known to have a strong correlation with increased morbidity and mortality [3–7]. Amputation may lead to social deprivation and great economic cost.

Several medical factors including atherosclerosis with multiple stenosis, nephropathy, and uncontrolled diabetes have been reported as predictive factors for amputation and mortality, which are the major outcomes of DFUs [2, 8]. However, socioeconomic factors may also importantly account for these outcomes because DFUs tend to occur as a result of chronic damage of tissues due to poor diabetes control. Diabetes, like other chronic diseases, is associated with socioeconomic position (SEP) [9–11]. Although the development of DFUs is associated with a low SEP [12], only a few reports exist on association between SEP and the prognosis of DFUs [13]. Patients with DFUs require additional comprehensive care with collaborations between primary and specialty care as well as more controlled health behavior. Furthermore, the cost associated with DFU treatment is high. A decreasing amputation rate among persons with diabetes has been reported, although a wide gap exists among societies and populations [14–17].

Equality in health care according to the need has been an important goal for health care policy in South Korea. All citizens of South Korea are covered by a mandatory health insurance system from birth to death. People are designated as National Health Insurance (NHI) or medical aid (MA) beneficiaries according to their insurance status. Most people who are covered under the NHI are associated with a low SEP and are recipients of the National Basic Livelihood Security System in South Korea. Understanding the association between amputation and mortality rate of DFUs with low SEP can lead to the development of health policies that can ameliorate the inequalities among the different SEPs.

This study aimed to determine the association between SEP and DFU outcomes, namely, amputation and mortality, and identify prognostic factors for these outcomes in a South Korean population.

## Methods

We investigated amputation and mortality rates among patients with DFUs in the South Korean population using the database of the National Health Insurance Sharing Service-National Sample Cohort (NHIS-NSC). The amputation and mortality rates based on residence, type of health insurance, type of medical institution, and income status were examined and the comorbidities associated with diabetes were also considered.

### Data collection

Data for the period 2011–2015 were extracted from the database of the NHIS-NSC, a population-based cohort established by the NHIS in South Korea. The NHIS collects data on the diagnosis, treatment, prescription, healthcare utilization, and inpatient and outpatient records [18]. Death records are merged from the Statistics Korea database along with aforementioned information and provided as claims data for research purposes [19]. NHIS database has been widely used for claims data-based studies, and its validity is described elsewhere [19, 20] [21].

The disease code for DFU was created in 2011 in South Korea. Until 2010, only disease codes indicating wounds such as gangrene and ulcers were used. Therefore, these patients with DFUs could have been overlooked and not identified. Therefore, to reduce bias, only data from 2011 onward was selected.

The cohort comprised 976,252 people (as of 2011, approximately 1.95% of the entire Korean population) who were randomly selected to represent the entire South Korean population. Using probability sampling, this random cohort sample was generated representing an individual’s total annual medical expenses within each stratum defined by age, sex, eligibility status (employed or self-employed), and income level (10 quartiles for each eligibility status and MA recipients) combinations using the proportional allocations of the 49,936,638 Korean residents in 2011.

### Operational definition

DFU was defined using the Korean Classification of Diseases-6th (KCD-6th) and KCD-7th versions for the period 2011–2012 and after 2013, respectively. Diabetic foot was defined using the KCD code, indicating diabetic foot complications (E1070–1072, E1170–1172, E1270–1272, E1370–1372, and E1470–1472). Patients with events within 1 year of the first diagnosis were excluded to reduce the possibility of reverse causation.

The data assessed in our study included demographic information such as age, sex, type of health insurance, residence regions, type of medical institutions at first diagnosis, household income, and other diabetes-related complications. The type of health insurance was divided into NHI and MA. The NHI covers approximately 96% of the total South Korean population, while the remaining 4% is covered by the MA program, which receives additional public assistance [22]. The hospitals were categorized as primary, secondary, and tertiary teaching hospitals and the areas of residence were divided into metropolitan and non-metropolitan areas. The causes of medical care utilization or death were recorded using the KCD-6th or KCD-7th classification systems. The study subjects were divided into six SEP groups based on insurance type, income level, and residence location (Group 1, MA and non-metropolitan; Group 2, MA and metropolitan; Group 3, low income NHI and non-metropolitan; Group 4, low income NHI and metropolitan; Group 5, high income NHI and non-metropolitan; and Group 6, high income NHI and metropolitan). High household income was defined as 8–10 deciles, while low household income was defined as 0–7 deciles.

The primary outcome was amputation, and the secondary outcome was mortality. Amputation was defined following the operation codes (N0572, N0573, N0574, N0575). The NHIS-NSC database was merged with death records and the cause of death data from the Statistics Korea database. Hypertensive disease (International Classification of Diseases, 10th Revision [ICD-10] codes I10–I15), coronary heart disease (ICD-10 codes I20–I25), cerebrovascular disease (ICD-10 codes I60–I69), and dyslipidemia (ICD-10 code E78) were considered comorbidities.

### Statistical analyses

Descriptive statistics are presented as *n* for dichotomous variables and means with standard deviations for continuous variables. Fisher’s exact test or Pearson’s χ^2^ test was used for categorical variables, and Student’s t-test was used for continuous variables. We used the Cox proportional-hazards model to evaluate the association of possible risk factors with amputation or mortality. Multivariate analysis was performed using Cox regression with factors that showed *p* < 0.1 in the univariate analyses. For censored cases, the follow-up duration was calculated from the date of the first diagnosis to the date of amputation or death. For cases not censored, we set the end of the follow-up point as the last date of medical care utilization (December 13, 2015). Amputation-free survival and overall survival were calculated using the Kaplan-Meier method. All statistical analyses were performed using SAS enterprise guide version 7.1 (SAS Inc., Cary, NC, USA) and R studio version 3.3.3 with *p* < 0.05 considered statistically significant.

### Ethical statement

The Institutional Review Board of Seoul National University Boramae Medical Center (No. 07–2019-25) granted this study an exemption with regards ethical approval. We did not obtain informed consent because the patients’ records and information were anonymized and de-identified before the analyses. The criteria of informed consent is approved by the the Institutional Review Board of Seoul National University Boramae Medical Center. All methods were carried out in accordance with relevant guidelines and regulations. The data used in this study is not publicly available.

## Results

### Baseline characteristics of the study subjects

**Fig. 1:**
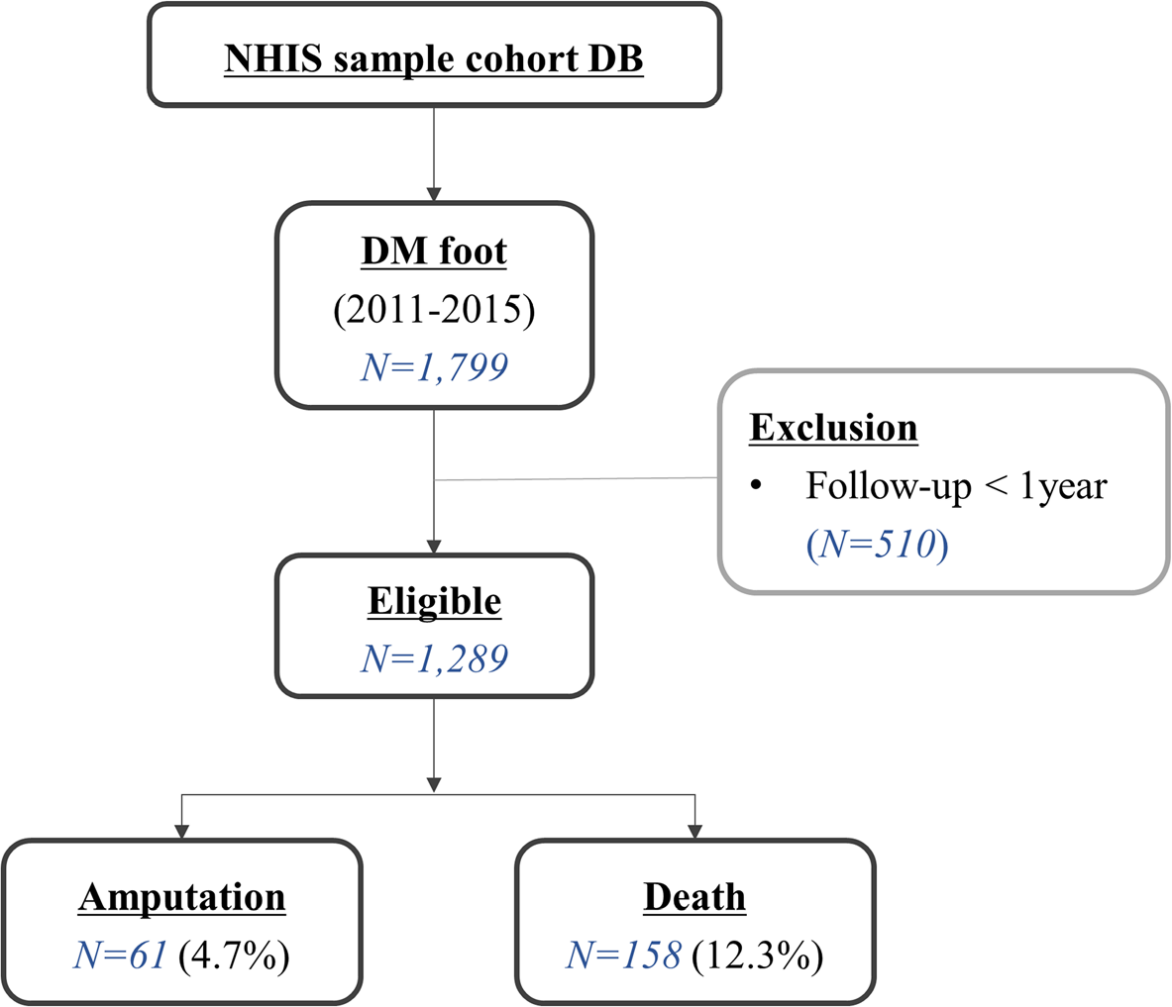
Flowchart of the selection process for the study population

A total of 1289 subjects were identified during the study period from the randomized cohort sample (Fig. 1). The mean age of the patients was 62.9 years, and 42.9% were females. With respect to health insurance status, 11.1% were MA beneficiaries while 88.9% were NHI beneficiaries. Among the NHI beneficiaries, 25.3% were in the bottom 20% of the income quartile. When classified by the level of medical institution, 11.9% were first diagnosed at tertiary teaching hospitals, 21.8% at general hospitals, and 66.3% at clinics (in South Korea, institutions with less than 30 beds are classified as clinic). Considering regional location, 82.9% resided in non-metropolitan areas. According to our SEP group definition, 6.6% were in group 1 (lowest SEP) and 15% were in group 6 (highest SEP). Detailed characteristics including comorbidities are listed in Table 1. A consort diagram is shown in Fig. 1.

**Table 1:** Baseline characteristics

Variable	Total (%) (***N*** = 1289)
Age (SD), years	62.89 (12.2)
Sex	
Male	720 (55.9)
Female	569 (44.1)
Follow-up (range), days	1214 [776–1606]
Type of health insurance	
NHI	1146 (89.0)
MA	143 (11.1)
Residence regions	
Metropolitan	220 (17.1)
Non-metropolitan	1069 (82.9)
Household income	
Low (0–7 decile)	689 (53.5)
High (8–10 decile)	449 (34.9)
Hypertension	1061 (82.3)
Dyslipidemia	1153 (89.4)
Stroke	526 (40.8)
Cardiovascular disease	677 (52.5)
Diabetic ophthalmopathy	268 (20.8)
Diabetic nephropathy	366 (28.4)
Diabetic neuropathy	628 (48.7)
Diabetic vasculopathy	681 (52.8)
Type of DM	
T1DM	101 (7.8)
T2DM	945 (73.3)
Unspecified DM	243 (18.9)
Type of institution	
Tertiary	154 (11.9)
Secondary	281 (21.8)
Primary	854 (66.2)
SEP group	
1	85 (6.6)
2	58 (4.5)
3	357 (27.7)
4	332 (25.8)
5	256 (19.9)
6	193 (15)

*SD* Standard deviation; *NHI* National health insurance; *MA* Medical aid; *DM* Diabetes mellitus; *SEP* Socioeconomic position

### Primary outcome: amputation

**Fig. 2:**
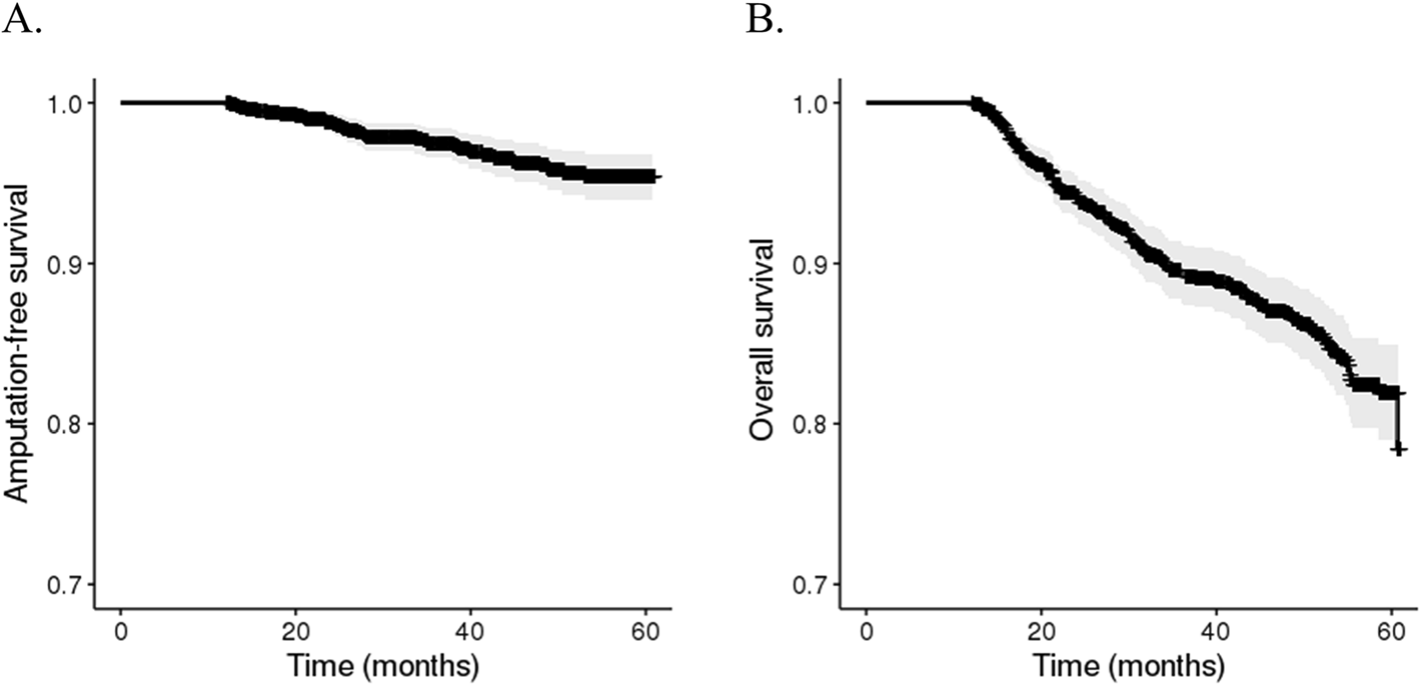
Kaplan-Meier plots for (**a**) amputation-free survival and (**b**) overall survival of patients with diabetic foot ulcers

**Fig. 3:**
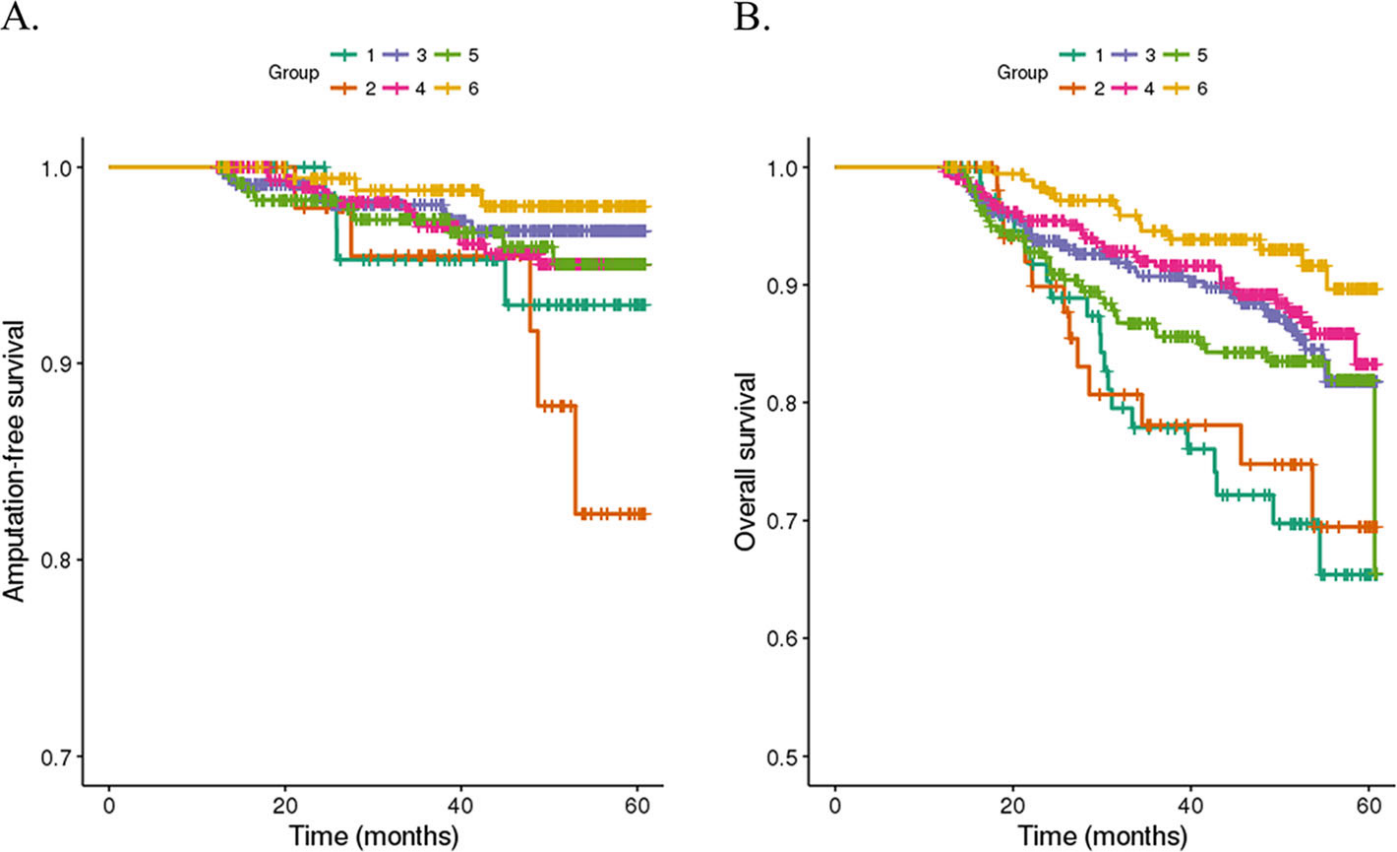
Kaplan-Meier plots for (**a**) amputation-free survival and (**b**) overall survival according to socioeconomic position group

**Fig. 4:**
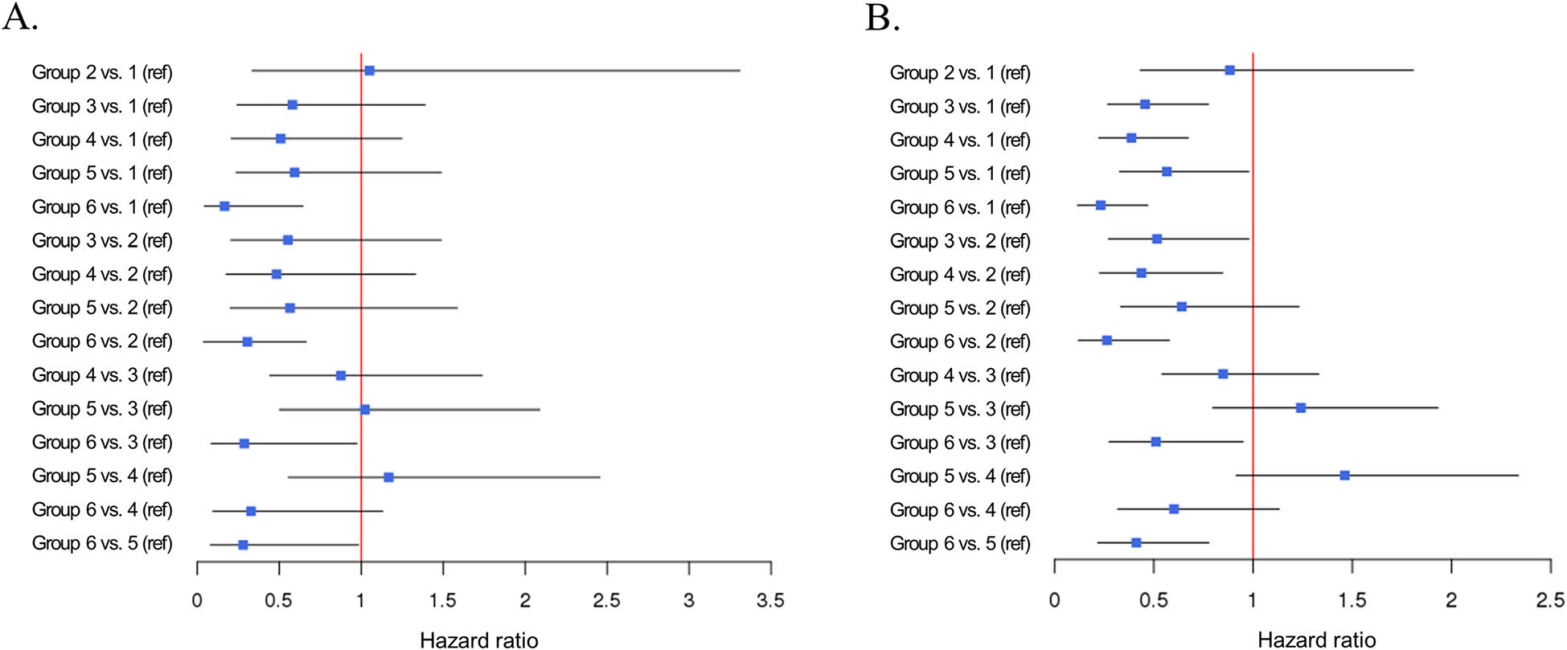
Forest plot showing hazard ratios of (**a**) amputation and (**b**) mortality for socioeconomic position group

Sixty-one patients (4.7%) underwent amputation during the follow-up duration of 656 days. Kaplan-Meier curves were generated for overall amputation-free survival (Fig. 2a), and the estimated 5-year amputation-free survival rate was 95.4%. The predictors for amputation are listed in Table 2. Male sex, ophthalmic complication (hazard ratio [HR], 1.89; 95% confidence interval [CI], 1.07–3.34), circulatory complications (HR, 2.14; 95% CI, 1.13–4.07), institution type (primary versus secondary general hospital: HR, 1.78; 95% CI, 1.01–3.12), and low SEP (highest versus lowest: HR, 5.13; 95% CI, 1.32–20.41) were independent predictors for amputation in DFU. The 5-year amputation-free survival rates differed among SEP groups; the 5-year survival rates were 93.0, 82.3, 96.8, 95.0, 95.0, and 98.0% in groups 1, 2, 3, 4, 5, and 6, respectively (Fig. 3a). HRs for amputation of each SEP group are shown in Fig. 4a.

**Table 2:** Cox regression results for Amputation

Variable	Univariable analysis				Multivariable analysis			
	HR	95% CI	p		HR	95% CI	p	
		Lower	Upper			Lower	Upper	
Age	0.998	0.977	1.019	0.840	–	–	–	–
Sex (ref = female)	1.184	0.862	1.627	0.005	2.412	1.355	4.292	0.003
SEP group				0.059				0.047
1 vs. 6 (ref)	6.000	1.551	23.208	0.009	5.140	1.319	20.002	0.018
2 vs. 6 (ref)	6.303	1.506	26.394	0.012	5.555	1.322	23.347	0.019
3 vs. 6 (ref)	3.484	1.026	11.829	0.045	3.252	0.955	11.071	0.059
4 vs. 6 (ref)	3.053	0.884	10.545	0.078	2.876	0.831	9.953	0.095
5 vs. 6 (ref)	3.566	1.016	12.515	0.047	3.260	0.926	11.482	0.066
Hypertension (ref = 0)	1.522	0.692	3.345	0.300	–	–	–	–
Stroke (ref = 0)	1.499	0.907	2.476	0.110	–	–	–	–
Cardiovascular disease (ref = 0)	1.531	0.912	2.569	0.110	–	–	–	–
Diabetic ophthalmopathy (ref = 0)	2.597	1.553	4.341	0.001	1.893	1.072	3.343	0.028
Diabetic nephropathy (ref = 0)	1.685	1.008	2.816	0.047	1.063	0.604	1.871	0.833
Diabetic neuropathy (ref = 0)	1.783	1.062	2.992	0.029	1.092	0.618	1.928	0.762
Diabetic vasculopathy (ref = 0)	2.788	1.557	4.993	< 0.001	2.141	1.127	4.066	0.020
Type of institution				0.048				0.068
Secondary vs. Primary (ref)	2.202	1.154	3.528	0.014	1.780	1.014	3.123	0.044
Tertiary vs. Primary (ref)	1.589	0.758	3.328	0.220	1.322	0.625	2.797	0.466

*SEP* Socioeconomic position; *ref*. Reference; *HR* Hazard ratio; *CI* Confidence interval

### Secondary outcome: death

In total, 158 patients (12.3%) died during the follow-up duration of 880 days. Kaplan-Meier curves were generated for overall survival (Fig. 2b). The estimated 5-year overall survival rate was 78.5%. Older age (HR, 1.06), ophthalmic complications (HR, 1.74; 95% CI, 1.21–2.49), circulatory complications (HR, 1.71; 95% CI, 1.15–2.52), institution type (primary versus secondary general hospital: HR, 1.84; 95% CI, 1.28–2.65), and low SEP (highest versus lowest: HR, 2.65; 95% CI, 1.28–5.52) were independent predictors of mortality in DFU. Independent predictors are listed in Table 3. The 5-year overall survival rates differed among the SEP groups; the 5-year survival rates were 65.4, 69.5, 81.8, 83.3, 65.5, and 89.6% in groups 1, 2, 3, 4, 5, and 6, respectively (Fig. 3b). HRs for death of each SEP group are shown in Fig. 4b.

**Table 3:** Cox regression results for Death

Variable	Univariable analysis				Multivariable analysis			
	HR	95% CI	p		HR	95% CI	p	
		Lower	Upper			Lower	Upper	
Age	1.058	1.042	1.075	< 0.001	1.057	1.039	1.073	< 0.001
Sex (ref = female)	1.184	0.862	1.627	0.300	–	–	–	–
SEP group				< 0.001				0.006
1 vs. 6 (ref)	4.289	2.132	8.621	< 0.001	3.336	1.649	6.749	0.001
2 vs. 6 (ref)	3.789	1.728	8.302	0.001	3.672	1.666	8.094	0.001
3 vs. 6 (ref)	1.956	1.052	3.637	0.034	2.192	1.174	4.091	0.014
4 vs. 6 (ref)	1.660	0.876	3.145	0.120	1.983	1.043	3.772	0.037
5 vs. 6 (ref)	2.425	1.239	4.575	0.006	2.251	1.182	4.283	0.013
Hypertension (ref = 0)	2.403	1.334	4.329	0.004	0.924	0.492	1.737	0.807
Stroke (ref = 0)	2.079	1.516	2.850	< 0.001	1.234	0.882	1.725	0.219
Cardiovascular disease (ref = 0)	2.147	1.532	3.008	< 0.001	1.275	0.879	1.849	0.199
Diabetic ophthalmopathy (ref = 0)	2.370	1.712	3.281	< 0.001	1.768	1.237	2.526	0.002
Diabetic nephropathy (ref = 0)	1.767	1.284	2.433	< 0.001	1.174	0.822	1.676	0.375
Diabetic neuropathy (ref = 0)	1.778	1.290	2.449	< 0.001	1.033	0.727	1.470	0.852
Diabetic vasculopathy (ref = 0)	2.523	1.777	3.582	< 0.001	1.668	1.129	2.463	0.010
Type of institution				0.005				0.008
Secondary vs. Primary (ref)	1.789	1.255	2.549	0.001	1.776	1.237	2.550	0.002
Tertiary vs. Primary (ref)	1.482	0.936	2.345	0.093	1.415	0.885	2.260	0.147

*SEP* Socioeconomic position; *ref*. Reference; *HR* Hazard ratio; *CI* Confidence interval

## Discussion

Our study results indicate that a low SEP, reflected by area residence, income level, and type of insurance, is associated with poor outcomes in patients with DFU. Both amputation and mortality rates showed significant differences between the lowest and highest SEP groups. This association was significant after considering age, sex, and comorbidities. Similarly, association between lower SEP and high rate of lower extremity amputation has been reported in peripheral vascular disease [23–26]. While the pathophysiology of peripheral vascular disease overlaps with DFUs, these studies are in line with our results. According to the institution type at first diagnosis, secondary hospitals showed a significantly higher risk than primary hospitals. This may imply that while the phase of referral to specialists in South Korea was performed without delay, patient-related factors such as SEP may have largely affected the prognosis.

We investigated factors representing biological mediators for amputation including neuropathy and vasculopathy, and disease severity of diabetes including nephropathy ophthalmopathy. Between the biological mediators, the main etiologies of DFU [27], vasculopathy had more significant impact on DFU outcomes. For the variables representing disease severity, ophthalmopathy but not nephropathy significantly predicted the DFU outcomes in multivariable analysis. Although ophthalmopathy and nephropathy are both considered as typical microvascular complication of diabetes, main pathophysiologic mechanism of ophthalmopathy is retinal ischemia associated with abnormal microvasculature while nephropathy has much more complicated pathophysiology besides microcirculation alterations; structural changes of glomerulus, mesangial expansion and tubulointerstitial fibrosis [28]. Also in line with our results, diabetic retinopathy has been reported to be the predictor for other diabetic complications including DFU [29–33]. These findings consistently suggest that microvascular abnormality in diabetes is the key component in predicting DFU outcomes.

We suggest that poor access to care, processes and quality of care, and health behaviors associated with patient education [9] are potential explanations for our results. Poor access to care may be related to a delayed diagnosis. A low SEP population may not be aware of their foot problem, which impedes their clinical visit. Even though the patient perceives their problem, lack of access to adequate primary and advanced care may result in a delay. Poor processes and quality of care may be related to inappropriate treatment. DFUs require early expert assessment and multidisciplinary management in various aspects including infection, peripheral ischemia, and peripheral neuropathy [6]. Close collaboration and timely referral are essential between primary physicians and specialties to improve outcomes [34]. Physicians without awareness of diabetic foot problems may not closely examine the wound, which delays the referral to specialties. Among patients who were referred to specialties, those with a low SEP had poor accessibility to frequent clinical visits because of cost or location. DFUs account for a large proportion of rising health care costs for persons with diabetes [35, 36]. This would cause difficulty in accessing care for patients with a low SEP. Sporadic clinical visits prevent frequent evaluations, impeding appropriate education and timely management. Delayed diagnosis, inappropriate treatment, and poor compliance worsen DFUs, increasing the risk of amputation and death.

The strength of this study is that its target sample group was randomly selected which can represent the total South Korean population. Although South Korea provides health insurance coverage for all citizens, blind spots exist, especially for diseases that require meticulous care. The findings from investigations into the prevalence of DFUs and the predictive factors for its prognosis, can guide the rational development of appropriate health care policies. According to our study, the low SEP group may be considered a high-risk group for poor DFU outcomes. While DFUs are associated with high health-related costs in persons with diabetes, patients with a low SEP are more likely to be neglected, which makes it a vicious cycle. DFUs, with amputation as the outcome, are associated with higher health-related costs caused by hospitalization, rehabilitation, home care, and social services for the disabled. Therefore, prevention of amputation through appropriate wound care can be the most important step for cost reduction in these patients. Management health programs will be useful for DFU patients with a low SEP, if provided by the society. Many studies have reported that prevention and screening programs for DFUs can reduce the rates of amputation, re-ulceration, and hospitalization [37–39]. Patout et al. [40] implemented a comprehensive prevention program for lower extremity amputations due to diabetes in Louisiana for low-income African-American populations. This resulted in a profound reduction in DFU-related complications including hospitalization, length of hospital stay, emergency room visits, foot operations, and lower extremity amputations after 1 year. According to a study conducted in the United Kingdom, developing DFU screening and preventive programs cost GB£100,000 (US$160,000) but saved approximately GB£3000 per amputation [41]. In this regard, diabetic foot management programs for DFU patients with a low SEP would largely reduce amputation and mortality rates, which can further reduce health-related costs. Regular foot inspections and education programs can be useful management tools. Education for primary care physicians may also be helpful [42]. The proportion of persons with diabetes undergoing annual foot examination in primary care hospitals is less than 49% [40]. Especially in South Korea, the proportion was even lower than 10% following the reports of 2007 [43]. In another study, of diabetic patients, who were admitted for foot infections, only 14% received fundamental foot examinations [44].

Our study has some limitations. First, our data did not include lifestyle, smoking, or health-related clinical factors such as hemoglobin A1c level and body mass index, which could have influenced the outcomes. Second, although the claims data were available from the year 2002, our study only targeted data from the year 2011 onwards because this was the year when the specific disease code for the diabetic foot was generated. This inevitably reduced the total sample size. Third, major and minor amputation was not separately considered but was analyzed together as amputation event. First amputation event, major or minor, has its value but cannot reflect the severity of the outcome. Forth, death was not investigated in a disease specific manner and further investigation would be required. Lastly, this is a population-based study conducted in South Korea, and its findings may not necessarily be generalizable to the people of other countries with different medical insurance systems. Future studies that consider a more comprehensive range of factors including medication, laboratory data, and lifestyles are recommended to validate the findings of this study.

## Conclusions

In conclusion, our results suggest that DFU patients with a low SEP are strongly associated with an increased rate of amputation and mortality. Hence, more attention needs to be given to such patients. Addressing SEP in patients with DFUs and providing targeted care for them may improve the prognosis.

## Data Availability

The datasets used and analysed during the current study are available from the corresponding author on reasonable request.

## Abbreviations

DFUs: Diabetic foot ulcers
SEP: Socioeconomic position
NHI: National health insurance
NHIS-NSC: National health insurance sharing service-national sample cohort
KCD: Korean classification of diseases
MA: Medical aid
ICD-10: International classification of diseases, 10th revision
HR: Hazard ratio
CI: Confidence interval
